# Nighttime Systolic Blood-Pressure Load Is Correlated with Target-Organ Damage Independent of Ambulatory Blood-Pressure Level in Patients with Non-Diabetic Chronic Kidney Disease

**DOI:** 10.1371/journal.pone.0131546

**Published:** 2015-07-17

**Authors:** Cheng Wang, Jun Zhang, Wenjie Deng, Wenyu Gong, Xun Liu, Zengchun Ye, Hui Peng, Tanqi Lou

**Affiliations:** Division of Nephrology, Department of Medicine, Third Affiliated Hospital of Sun Yat-Sen University, Guangzhou, Guangdong 510630, China; University of Perugia, ITALY

## Abstract

**Background:**

The impacts of blood pressure (BP) load on target-organ damage in patients with chronic kidney disease (CKD) are largely unclear. We examined whether BP load is correlated with target-organ damage (TOD) in Chinese CKD patients independent of BP level.

**Methods:**

We recruited 1219 CKD patients admitted to our hospital division in this cross-sectional study. The TOD were measured by estimated glomerular filtration rate (eGFR), proteinuria, left ventricular mass index (LVMI) and carotid intima-media thickness (cIMT) in this study. Univariate and multivariate linear analyses were used to evaluate the relationship between systolic blood pressure (SBP) load, diastolic blood pressure (DBP) load and these renal, cardiovascular parameters.

**Results:**

In multivariable-adjusted models, BP load and ambulatory BP levels both independently correlated with LVMI, eGFR and proteinuria in all groups of CKD patients (p<0.05), 24-h SBP correlated with cIMT only in non-diabetic CKD patients without hypertension (p<0.05), while nighttime SBP load was associated with cIMT only in non-diabetic CKD patients (p<0.05). Furthermore, nighttime SBP load additionally increased coefficient of determination (R^2^) and correlated with LVMI, proteinuria in non-diabetic CKD patients without hypertension (R^2^ = 0.034, P<0.001 and R^2^ = 0.012, P = 0.006 respectively) and LVMI, cIMT, eGFR in non-diabetic CKD patients with hypertension (R^2^>0.008, P<0.05) in multivariable-adjusted model which already including the 24-h BP. BP load did not refine this correlation based on the 24-h BP level in diabetic CKD patients.

**Conclusion:**

Night-time SBP load was correlated with TOD in patients with non-diabetic chronic kidney disease independent of BP level.

## Background

Chronic kidney disease (CKD) has become an important public-health issue in China. The number of patients with CKD in China is ≈119.5 million based on the fact that the prevalence of CKD in China is 10.8% [1]. Hypertension is the most prevalent independent risk factor for the progression of renal failure and cardiovascular disease [2]. Lowering blood pressure (BP) can delay the progression of CKD [3]. It is very important to assess BP accurately and completely in CKD patients.

Over the last decade, ambulatory blood pressure monitoring (ABPM) employing a wearable, oscillometric BP device to automatically measure and record BPs at prescribed intervals has emerged as a valuable tool for assessing BP. ABPM provides additional information on the mean BP (systolic and diastolic), BP load (proportion of BP readings above a threshold BP), and non-dipping (<10% decrease in night-time BP relative to daytime BP). There are many trials on ABPM and the dipper pattern [4, 5], whereas studies on BP load are very limited especially for adult patients. BP load has been suggested to be a clinically useful parameter complementing quantification of the corresponding average BP levels, which might be an index of hemodynamic burden on the cardiovascular system in addition to the information carried by average BP levels. A series of studies have set the role of BP load in subclinical organ damage in hypertensive patients: daytime systolic blood pressure (SBP) load was an independent predictor of the left ventricular mass index (LVMI) [6], night-time SBP load to have a stronger association with the LVMI [7], while diastolic blood pressure (DBP) load was an independent predictor of intima-media thickness [8]. All these studies on BP load and target-organ damage (TOD) were limited by small sample size as well as methodological problems related to ABPM and short-term follow-up, which might lead to inconsistent conclusions [9,10]. The relationship between BP load and TOD dependent on BP levels is controversial: one study involving 130 hypertensive patients showed that high 24-h SBP load might be associated, independently of the average level of 24-h SBP, with an adverse cardiovascular risk [8] while another study involving 869 individuals without therapy showed that BP load was associated with target-organ damage, but not independently of BP level [11].

The role of BP load in patients with CKD is not known. We previously found that TOD such as estimated glomerular filtration rate (eGFR), proteinuria, LVMI and carotid intima-media thickness (cIMT) were significantly related to the parameters of AMBP in Chinese CKD patients [12]. We hypothesized that BP load correlated with TOD in Chinese CKD patients based on the data stated above. We carried out this cross-sectional study to ascertain if different times of BP load were associated with TOD, and whether such an association survives after accounting for the impact of average BP levels in Chinese CKD patients.

## Subjects, Materials and Methods

### Study population

The study protocol was approved by the ethics committee of the Third Hospital of Sun Yat-Sen University (Guangdong, China). This cross-sectional study were from May 2010 to October 2014, all study participants provided written informed consent to be included in the study.

Inclusion criteria were: 1) consecutive CKD patients who had not undergone any type of antihypertensive treatment within 1 month; 2) pre-dialysis CKD patients with a stable disease state. Exclusion criteria were: 1) undergoing treatment with corticosteroids or hormones; 2) acute changes in the eGFR >30% in the previous three months; pregnancy; 3) history of abuse of drugs or alcohol; night work or shift-work employment; 4) acquired immunodeficiency syndrome; 5) cardiovascular disorders (unstable angina pectoris, heart failure, life-threatening arrhythmia, atrial fibrillation and grade III–IV retinopathy); 6) intolerance to ABPM; 7) inability to communicate and comply with all of the study requirements; 8) maintenance dialysis. Finally, 1219 CKD patients were enrolled in this study.

### Ambulatory blood pressure monitoring

Patients underwent 24-h ABPM using a TM-2430 Monitor (A&D, Tokyo, Japan). Cuff size was chosen based on arm circumference and fixed to the non-dominant arm. BP readings were obtained in the morning at three points from 7 am to 10 pm using a mercury sphygmomanometer by a physician who did not have access to ABP values. Then BP was recorded every 15 min from 7 am to 10 pm, and every 30 min from 10 pm to 7 am. Monitoring was done on a working day. Patients were asked to attend to their usual activities but to keep motionless at the time of measurement. Patients had no access to ABP values. Strenuous physical activity was discouraged in all patients during the monitoring period, and their daily activities were comparable. BP series were eliminated from the analyses if: >30% of the measurements were lacking; they had missing data for >3-h spans; they were collected from subjects who were experiencing an irregular rest–activity schedule or a night-time sleep span <6 h or >12 h during monitoring. Night time BP was defined as the average BP from the time of patients went to bedtime until the time they got out of bed, and daytime as the average BPs recorded during the rest of the day.

#### Cardiac assessment

Cardiac structure was assessed by two investigators trained for this purpose before starting the study. Left ventricular mass (LVM) were assessed using two-dimensional echocardiography. Linear measurements of end-diastolic interventricular septal wall thickness (IVSd), end-diastolic left ventricular internal dimension (LVIDd), and end-diastolic posterior wall thickness (PWTd) were obtained from M-mode tracings. LVM was calculated using the formula [13], and the left ventricular mass index (LVMI) was obtained by calculating the ratio of LVM to body surface area [14].

#### Carotid ultrasonography

Carotid intima-media thickness (cIMT) was assessed by two trained investigators before study commencement. A SonoSite MicroMaxx Ultrasound System paired with a 5–10-MHz Multifrequency High-resolution linear transducer (Bothell, WA, USA) with Sono-Calc IMT software was used for taking automatic measurements of cIMT. This was achieved by averaging three measurements taken on each carotid artery (anterior, lateral and posterior directions) and measuring the distance between the leading edge of the lumen–intima interface and the leading edge of the collagenous upper layer of the adventitia using high-resolution B-mode ultrasonography.

#### Renal assessment

Kidney damage was assessed by measuring the serum concentrations of creatinine, which is measured by the enzymatic method, traceable to the isotope dilution mass spectrometry. The estimated glomerular filtration rate (eGFR) was calculated using a modified version of the Modification of Diet in Renal Disease (MDRD) equation based on data from Chinese CKD patients[15].

#### Other data collection

We collected urine samples from 7 am to 7 am the next day to detect the extent of proteinuria and sodium levels over 24 h. These patients were asked to void their bladders at 7 am. Proteinuria was measured by immunoturbidimetry. In addition, medical history, including demographic and laboratory data [hemoglobin, albumin, globulin, calcium, phosphate, intact parathyroid hormone (iPTH), serum fasting glucose, cholesterol, triglycerides (TGs), high-density lipoprotein-cholesterol (HDL-C), low-density lipoprotein-cholesterol (LDL-C), homocysteine, uric acid, serum cystatin C, blood urea nitrogen (BUN)] were obtained at the initial study visit. All these experimental data were measured using a 7180 Biochemistry Auto-analyzer (Hitachi, Tokyo, Japan).

## Definitions

The diagnosis of hypertension was based on accepted criteria for ABPM [16]: Ambulatory hypertension was defined as mean 24-h BP >130/80 mmHg for SBP/DBP. “Daytime” and “night-time” were defined as the clock time intervals from 7 am to 10 pm and from 10 pm to 7 am, respectively. BP load was the percentage of BP values reaching or exceeding 135 mmHg systolic or 85 mmHg diastolic during day time, or 120 mmHg systolic or 70 mmHg diastolic during night-time [9]. Studies found that SBP load values >50% were correlated significantly with TOD, and values of SBP load >50% might be used to identify severe ambulatory hypertension at risk of TOD [16, 17]. Hence, we used 50% as the cutoff point when we undertook analyses, and patients with BP load >50% were said to have an abnormal BP load.

CKD was defined as the presence of kidney damage or decreased renal function (eGFR of <60 mL/min per 1.73 m^2^) for ≥3 months according to guidelines set by the Kidney Disease Outcomes Quality Initiative [18].

Diabetes mellitus (DM) was defined as the need for anti-diabetic drugs or meeting the diagnostic criteria for DM specified by chinese guidelines for diabetes prevention and treatment [19]

### Statistical analyses

Continuous variables are the mean ± standard deviation (SD). Frequency distributions were used for qualitative variables. Non-parametric variables are expressed as median and interquartile ranges. Log transformation for the eGFR was done in view of the skewed distribution of these data. Comparisons for continuous variables between groups were tested by the Student’s *t* test or non-parametric test. Differences among categorical variables were analyzed using the χ^2^ test or the two-tailed Fisher’s exact test, as appropriate. The relationship between two continuous variables was assessed by a bivariate correlation method (Pearson’s correlation).

Univariate linear regression models (enter method) were used to study the association of TOD (LVMI, cIMT, log eGFR, and log proteinuria) and BP load (daytime SBP load and DBP load, night-time SBP load and DBP load), DM (1 = no, 2 = yes), triglycerides (TG), low-density lipoprotein-cholesterol (LDL-C), cholesterol, hemoglobin, albumin (ALB), eGFR, calcium, phosphorus, body mass index (BMI), 24-h SBP and 24-h DBP respectively. To further study the association of 24-BP level, BP load and TOD, multivariate linear regression analysis were performed (stepwise method) which adjusted for age, sex (1 = female, 2 = male), current smoker (1 = no, 2 = yes), alcohol intake (1 = no, 2 = yes) and those variables with a significant association with a dependent variable in the univariate linear regression. In the next step of the analysis, we added BP load (only the BP load which P<0.05 in model 2) to model 3 which already including the 24-h BP (only the BP which P<0.05 in model 2) and other covariables. We tested whether BP load was able to additionally explain the variance of TOD. All values are two-tailed. p<0.05 was considered significant. Data were analyzed using SPSS v18.0 (IBM, Armonk, NY, USA)

## Results

### Baseline characteristics of CKD patients

A total of 1219 CKD patients were enrolled in this study. The mean age was 44.0±16.6. The male:female ratio was 723:496, which comprised 432 non-diabetic CKD patients without hypertension, 565 non-diabetic CKD patients with hypertension and 222 diabetic patients (18.2%).

Hypertensive non-diabetic CKD patients were older, a higher percentage of smokers and alcohol consumers, higher BMI, higher 24h BP and BP load, higher fasting glucose, cholesterol, LDL-C, calcium, phosphate, creatinine, PTH levels, lower eGFR and hemoglobin, higher LVMI and cIMT compared with non-diabetic CKD patients without hypertension(p<0.05). While diabetic patients were older, a higher percentage of smokers and alcohol consumers, higher BMI, higher 24h BP and BP load, higher fasting glucose, cholesterol, LDL-C, calcium, phosphate, creatinine, PTH levels, lower eGFR and hemoglobin, higher LVMI and cIMT (p<0.05) compared with non-diabetic CKD patients without hypertension(p<0.05). Compared with diabetic CKD patients with hypertension, diabetic patients were older and had: higher BMI, higher BP load and 24h DBP levels, higher fasting glucose, cholesterol, LDL-C, creatinine, PTH levels, lower eGFR and hemoglobin, higher cIMT (p<0.05) compared with non-diabetic CKD patients without hypertension(p<0.05) (Table 1).

**Table 1:** Baseline characteristics in study participants.

Variables	Non-diabetic patients without hypertension	Non-diabetic patients with hypertension	Diabetic patients
	n = 432	n = 565	n = 222
Age(years)	35.9±14.9	44.7±15.9 *	57.7±11.6 * ^**†**^
Men/Women	238/194	346/219	139/83
Current smoker, n (%)	69(16)	120(21.2) *	50(22.5) *
Alcohol intake, n (%)	27(6.3)	61(10.8) *	28(12.6) *
BMI(kg/m^2^)	22.1±3.4	23.1±3.5 *	24.4±3.2 * ^**†**^
24-h SBP (mmHg)	115.2.2±8.2	144.1±13.2 *	144.6±17.7 *
24-h DBP (mmHg)	70.1±5.4	86.1±8.9 *	80.5±8.1 * ^**†**^
Daytime SBP load (%)	10.5(5.2–16.1)	58.8(35.2–84.6) *	67.2(35.0–88.3) * ^**†**^
Daytime DBP load (%)	7.5(3.2–12.5)	36.7(18.4–63.6) *	10.4(18.4–36.4) * ^**†**^
Nighttime SBP load (%)	13.3(5.6–33.3)	88.9(66.7–100.0) *	94.4(75.0–100.0) * ^**†**^
Nighttime DBP load (%)	22.2(6.2–47.1)	87.6(71.7–94.6) *	77.8(52.9–94.1) * ^**†**^
Proteinuria (g/24 h)	0.86(0.30–3.20)	1.4(0.6–3.0) *	1.9 (0.7–4.1) * ^**†**^
Hemoglobin (G/L)	126.2±23.9	108.1±31.3 *	102.4±24.6 * ^**†**^
Albumin (G/L)	33.4±9.6	34.0±8.1	34.9±6.2
Fasting Glucose(mmol/L)	4.8 ± 1.1	4.9±1.0 *	6.9±3.2 * ^**†**^
Cholesterol (mmol/l)	6.2±3.1	5.8±2.9 *	5.1±1.8 * ^**†**^
Triglyceride(mmol/L)	1.5(1.0–2.3)	1.7(1.1–2.7)	1.5(1.1–2.4)
LDL-C(mmol/L)	4.1±2.5	3.7±2.2 *	3.2±1.4 * ^**†**^
Ca(mg/dl)	8.6±0.9	8.5±1.0 *	8.7±0.8
P (mmol/L)	1.3±0.2	1.5±0.6 *	1.5±0.5 *
PTH	40.5(28.2–71.3)	87.2(43.3–275.2) *	112.2(41.6–243.2) * ^**†**^
Serum creatinine (umol/l)	90.0(67.0–151.7)	288.8(113.8–692.0) *	309.8(137.0–595.3) * ^**†**^
eGFR (ml/min per 1.73m2)	86.8(37.5–120.1)	19.6(6.2–60.2) *	17.0(6.6–49.3) * ^**†**^
LVMI (g/m^2^)	85.2±21.5	114.8±35.8 *	120.8±33.6 *
cIMT(mm)	0.61±0.19	0.71±0.25 *	0.94±0.41 * ^**†**^

(BMI: body mass index; cIMT: carotid intima-media thickness; BMI: body mass index; DM: diabetes mellitus; eGFR: estimated glomerular filtration rate; LDL-C: low-density lipoprotein cholesterol; LVMI: left ventricular mass index; iPTH: intact parathyroid hormone; SBP: systolic blood pressure; DBP: diastolic blood pressure); [calcium] (mg/dL) = measured [calcium] + (4.0-[serum albumin (mg/dl)]) × 0.8.

* indicates control with Non-diabetic patients without hypertension, p<0.05.

^†^ indicates control with Non-diabetic patients with hypertension group, p<0.05.

### Correlation between BP load and TOD in CKD patients

Univariate correlation analyses showed that daytime SBP load, night-time BP load and 24h BP levels correlated with LVMI, cIMT and eGFR in non-diabetic CKD patients without hypertension (p<0.05), while only 24h SBP, rather than BP load, was associated with proteinuria in these patients. BP load and 24h BP levels were significantly related to LVMI and eGFR (p<0.05), SBP load and 24h SBP levels correlated with cIMT (p<0.05), while no correlation was found between BP load and proteinuria in non-diabetic CKD patients with hypertension (Table 2).

**Table 2:** Associations between BP load and organ damage in Non-diabetic patients.

	Without hypertension				With hypertension			
	LVMI	cIMT	Lg (eGFR)	Lg (Pro)	LVMI	cIMT	Lg (eGFR)	Lg (Pro)
Daytime SBP load (%)	0.224**	0.180*	-0.196**	0.061	0.373**	0.204**	-0.339**	0.028
Daytime DBP load (%)	0.097	0.117	-0.053	0.005	0.226**	0.003	-0.201**	0.057
Nighttime SBP load (%)	0.424**	0.335**	-0.288**	0.077	0.339**	0.196**	-0.334**	0.063
Nighttime DBP load (%)	0.447**	0.300**	-0.343**	-0.009	0.242**	0.003	-0.269**	0.044
24-h SBP (mmHg)	0.277**	0.312**	-0.152**	0.112*	0.402**	0.184**	-0.349**	0.005
24-h DBP (mmHg)	0.229**	0.256**	-0.196**	0.028	0.232**	0.004	-0.224**	0.063

(cIMT: carotid intima-media thickness; Lg(eGFR): Lg transformation for estimated glomerular filtration rate; Lg(Pro): Lg transformation for proteinuria; LVMI: left ventricular mass index SBP: systolic blood pressure; DBP: diastolic blood pressure)

* indicates p<0.05.

** indicates p<0.01.

SBP load and 24h SBP correlated with LVMI (p<0.05), daytime SBP load, nighttime BP load and 24h SBP correlated with eGFR (p<0.05), BP load and 24h BP levels correlated with proteinuria (p<0.05), while no correlation was found between BP load and cIMT in diabetic patients (Table 3).

**Table 3:** Associations between BP load and organ damage in Diabetic patients.

	LVMI	cIMT	Lg(eGFR)	Lg(Pro)^ ^
Daytime SBP load (%)	0.325**	-0.051	-0.266**	0.220**
Daytime DBP load (%)	-0.091	-0.154	-0.059	0.169*
Nighttime SBP load (%)	0.242**	-0.102	-0.278**	0.222**
Nighttime DBP load (%)	0.107	-0.119	-0.191**	0.193**
24-h SBP (mmHg)	0.330**	-0.088	-0.299**	0.253**
24-h DBP (mmHg)	0.076	-0.135	-0.090	0.233**

(cIMT: carotid intima-media thickness; Lg(eGFR): Lg transformation for estimated glomerular filtration rate; Lg(Pro): Lg transformation for proteinuria; LVMI: left ventricular mass index SBP: systolic blood pressure; DBP: diastolic blood pressure)

* indicates p<0.05.

** indicates p<0.01.

### Associations of TOD with BP Level or BP Load

In multivariable-adjusted analysis, model 1 without BP load inclusion: 24-h SBP was associated with LVMI, cIMT, proteinuria, while 24-h DBP correlated with eGFR in non-diabetic CKD patients without hypertension. model 2 without 24-h BP inclusion: nighttime SBP load correlated with LVMI, cIMT, proteinuria, while nighttime DBP correlated with eGFR in these patients. Model 3 with aforementioned covariables and both 24h-BP and BP load (p<0.05) in model 1 and model 2 inclusion, nighttime SBP load was able to additionally explain the variance of LVMI and proteinuria in these patients (R^2^ = 0.034, P<0.001 and R^2^ = 0.012, P = 0.006 respectively) (Table 4).

**Table 4:** Multivariate linear regression analysis between 24h-BP, BP load and organ damage respectively in non-diabetic patients without hypertension.

	LVMI â (95%CI)	cIMT â (95%CI)	Lg(eGFR) â (95%CI)	Lg(Pro) â (95%CI)
Model 1				
24-h SBP	0.187 (0.217–0.744) **	0.129 (0.000–0.006) *	-	0.076(0.001–0.009)*
24-h DBP	-	-	-0.083 (-0.011–0.002) **	-
Model 2				
Daytime SBP load (%)	-	-	-	-
Daytime DBP load (%)	-	-	-	-
Nighttime SBP load (%)	0.224 (0.101–0.364) **	0.178(0.000–0.003) *	-	0.137(0.001–0.004)**
Nighttime DBP load (%)	-	-	-0.082 (-0.002–0.000) *	
Model 3				
Daytime SBP load (%)	-	-	-	-
Daytime DBP load (%)	-	-	-	-
Nighttime SBP load (%)	0.238 (0.105–0.355)**	0.055 (-0.001–0.002)	-	0.144(0.001–0.004)*
Nighttime DBP load (%)	-	-	-0.071 (-0.003–0.000)	-
R^2,^ %	0.034	0.002	0.001	0.012
P Value	<0.001	0.424	0.300	0.006

(age, sex(female/male), Current smoker(N/Y), Alcohol intake(N/Y), ALB, LDL-C, Cholesterol, Hemoglobin, Ca, P, eGFR were adjusted to analysis the relationship between LVMI and 24h-BP (model 1) and the relationship between LVMI and BP load (model 2); Age, sex(female/male), Current smoker(N/Y), Alcohol intake(N/Y), ALB, eGFR, BMI were adjusted to analysis the relationship between cIMT and 24h-BP (model 1) and the relationship between cIMT and BP load (model 2); Age, sex(female/male), Current smoker(N/Y), Alcohol intake(N/Y), ALB, LDL-C, Cholesterol, Hemoglobin, Ca, P were adjusted to analysis the relationship between eGFR and 24h-BP (model 1) and the relationship between eGFR and BP load (model 2); Age, sex(female/male), Current smoker(N/Y), Alcohol intake(N/Y), LDL-C were were adjusted to analysis the relationship between proteinuria and 24h-BP (model 1) and the relationship between proteinuria and BP load (model 2). Model 3 include the aforementioned covariables and both 24h-BP and BP load which p<0.05 in model 1 and model 2. R^2^ and P value indicates the R^2^ change and significance respectively comparing the model including the 24-h BP (p<0.05 in model 1) and covariables and a model additionally including BP load (p<0.05 in model 2).

(BMI: body mass index; CI, confidence interval; CIMT: carotid intima-media thickness; DM: diabetes mellitus; SBP: systolic blood pressure; DBP: diastolic blood pressure; Lg(eGFR): Lg transformation for estimated glomerular filtration rate; Lg(Pro): Lg transformation for proteinuria; iPTH: intact parathyroid hormone; LDL-C: low-density lipoprotein cholesterol; LVMI: left ventricular mass index.)

* indicates p<0.05.

** indicates p<0.01.

In multivariable-adjusted analysis, model 1 without BP load inclusion: 24-h SBP was associated with LVMI, while 24-h DBP correlated with eGFR and proteinuria in non-diabetic CKD patients with hypertension. model 2 without 24-h BP inclusion: SBP load correlated with LVMI, nighttime SBP load correlated with cIMT, nighttime BP load is associated with eGFR, while nighttime DBP load correlated with proteinuria in these patients. Model 3 with aforementioned covariables and both 24h-BP and BP load (p<0.05) in model 1 and model 2 inclusion, nighttime SBP load was able to additionally explain the variance of LVMI, cIMT and eGFR in these patients (R^2^ = 0.008, P = 0.037, R^2^ = 0.016, P = 0.011 and R^2^ = 0.009, P < 0.001 respectively (Table 5).

**Table 5:** Multivariate linear regression analysis between 24h-BP, BP load and organ damage respectively in non-diabetic patients with hypertension.

	LVMI â (95%CI)	cIMT â (95%CI)	Lg(eGFR) â (95%CI)	Lg(Pro) â (95%CI)
Model 1				
24-h SBP	0.309 (0.528–1.109) **	-	-	-
24-h DBP	-	-	-0.115(-0.010–-0.004) **	0.088 (0.001–0.006)*
Model 2				
Daytime SBP load (%)	0.182 (0.113–0.378)**	-	-	-
Daytime DBP load (%)	-	-	-	-
Nighttime SBP load (%)	0.226(0.088–0.212) **	0.178 (0.000–0.003) *	-0.084(-0.003–-0.001) *	-
Nighttime DBP load (%)	-	-	-0.082(-0.003–-0.001) *	0.074 (0.000–0.002)**
Model 3				
Daytime SBP load (%)	0.029 (-0.172–0.251)	-	-	-
Daytime DBP load (%)	-	-	-	-
Nighttime SBP load (%)	0.124 (0.035–0.316)*	0.178 (0.000–0.003) *	-0.080 (-0.003–-0.001) *	-
Nighttime DBP load (%)	-	-	-0.053 (-0.003–-0.000)	0.086 (-0.001–0.003)
R^2,^ %	0.008	0.016	0.009	0.000
P Value	0.037	0.011	< 0.001	0.735

(age, sex(female/male), Current smoker(N/Y), Alcohol intake(N/Y), ALB, LDL-C, Cholesterol, Hemoglobin, Ca, P, eGFR were adjusted to analysis the relationship between LVMI and 24h-BP (model 1) and the relationship between LVMI and BP load (model 2); Age, sex(female/male), Current smoker(N/Y), Alcohol intake(N/Y), ALB, eGFR, BMI were adjusted to analysis the relationship between cIMT and 24h-BP (model 1) and the relationship between cIMT and BP load (model 2); Age, sex(female/male), Current smoker(N/Y), Alcohol intake(N/Y), ALB, LDL-C, Cholesterol, Hemoglobin, Ca, P were adjusted to analysis the relationship between eGFR and 24h-BP (model 1) and the relationship between eGFR and BP load (model 2); Age, sex(female/male), Current smoker(N/Y), Alcohol intake(N/Y), LDL-C were were adjusted to analysis the relationship between proteinuria and 24h-BP (model 1) and the relationship between proteinuria and BP load (model 2). Model 3 include the aforementioned covariables and both 24h-BP and BP load which p<0.05 in model 1 and model 2. R^2^ and P value indicates the R^2^ change and significance respectively comparing the model including the 24-h BP (p<0.05 in model 1) and covariables and a model additionally including BP load (p<0.05 in model 2).

(BMI: body mass index; CI, confidence interval; CIMT: carotid intima-media thickness; DM: diabetes mellitus; SBP: systolic blood pressure; DBP: diastolic blood pressure; Lg(eGFR): Lg transformation for estimated glomerular filtration rate; Lg(Pro): Lg transformation for proteinuria; iPTH: intact parathyroid hormone; LDL-C: low-density lipoprotein cholesterol; LVMI: left ventricular mass index.)

* indicates p<0.05.

** indicates p<0.01.

In multivariable-adjusted analysis, model 1 without BP load inclusion: 24-h SBP was associated with LVMI, eGFR and proteinuria in diabetic CKD patients; model 2 without 24-h BP inclusion: daytime SBP load correlated with LVMI and proteinuria, while nighttime SBP load correlated with eGFR; model 3 with aforementioned covariables and both 24h-BP and BP load (p<0.05) in model 1 and model 2 inclusion, the inclusion of BP load cannot additionally explain the variance of LVMI, cIMT, proteinuria and eGFR in these patients (P>0.05) (Table 6).

**Table 6:** Multivariate linear regression analysis between 24h-BP, BP load and organ damage respectively in diabetic patients group.

	LVMI â (95%CI)	cIMT â (95%CI)	Lg(eGFR) â (95%CI)	Lg(Pro) â (95%CI)
Model 1				
24-h SBP	0.223 (0.119–0.762) **	-	-0.174(-0.008–-0.001) **	0.274 (0.002–0.007) **
24-h DBP	-	-	-	-
Model 2				
Daytime SBP load (%)	0.211(0.063–0.405) **	-	-	0.264 (0.001–0.004) **
Daytime DBP load (%)	-	-	-	-
Nighttime SBP load (%)	-	-	-0.143 (-0.004–0.000) *	-
Nighttime DBP load (%)	-	-	-	-
Model 3				
Daytime SBP load (%)	0.093 (-0.308–0.515)	-	-	-0.116 (-0.004–0.002)
Daytime DBP load (%)	-	-	-	-
Nighttime SBP load (%)	-	-	-0.026 (-0.004–0.003)	-
Nighttime DBP load (%)	-	-	-	-
R^2,^ %	0.001	-	0.000	0.006
P Value	0.621	-	0.789	0.245

(age, sex(female/male), Current smoker(N/Y), Alcohol intake(N/Y), ALB, LDL-C, Cholesterol, Hemoglobin, Ca, P, eGFR were adjusted to analysis the relationship between LVMI and 24h-BP (model 1) and the relationship between LVMI and BP load (model 2); Age, sex(female/male), Current smoker(N/Y), Alcohol intake(N/Y), ALB, eGFR, BMI were adjusted to analysis the relationship between cIMT and 24h-BP (model 1) and the relationship between cIMT and BP load (model 2); Age, sex(female/male), Current smoker(N/Y), Alcohol intake(N/Y), ALB, LDL-C, Cholesterol, Hemoglobin, Ca, P were adjusted to analysis the relationship between eGFR and 24h-BP (model 1) and the relationship between eGFR and BP load (model 2); Age, sex(female/male), Current smoker(N/Y), Alcohol intake(N/Y), LDL-C were were adjusted to analysis the relationship between proteinuria and 24h-BP (model 1) and the relationship between proteinuria and BP load (model 2). Model 3 include the aforementioned covariables and both 24h-BP and BP load which p<0.05 in model 1 and model 2. R^2^ and P value indicates the R^2^ change and significance respectively comparing the model including the 24-h BP (p<0.05 in model 1) and covariables and a model additionally including BP load (p<0.05 in model 2).

(BMI: body mass index; CI, confidence interval; CIMT: carotid intima-media thickness; DM: diabetes mellitus; SBP: systolic blood pressure; DBP: diastolic blood pressure; Lg(eGFR): Lg transformation for estimated glomerular filtration rate; Lg(Pro): Lg transformation for proteinuria; iPTH: intact parathyroid hormone; LDL-C: low-density lipoprotein cholesterol; LVMI: left ventricular mass index.)

* indicates p<0.05.

** indicates p<0.01.

## Discussion

The present study showed both ambulatory blood pressure and BP load were independent associated with TOD in CKD patients, furthermore, nighttime SBP load was able to additionally explain the variance of TOD in non-diabetic CKD patients. In this regard, nighttime BP load correlated with TOD in non-diabetic CKD patients was not fully dependent 24-h BP levels. According to our best knowledge, this is the first study to show the relationship between BP load and cardiac, large vascular, and renal damage in CKD patients. Our study suggested that nighttime SBP load might play a role in non-diabetic CKD patients.

One study from 8711 subject showed that BP load did not improve risk stratification based on 24-hour BP level even in patients without hypertension [20]. However, there are no data on the relationship between BP load and TOD in CKD patients dependent on the BP level or not. CKD patients are considered to be the “highest risk group” for subsequent cardiovascular vascular disease (CVD) events, and treatment recommendations based on stratification of CVD risk should take into account the highest-risk status [21]. We could not speculate any conclusion based on results from the general population, even patients with hypertension. In this study, we enrolled more than 1000 CKD patients, separated patients with hypertension and diabetic mellitus or not, and undertook a comprehensive assessment of these patients. We found that nighttime SBP load might be a new risk factor, independent of BP levels, for TOD in non-diabetic CKD patients, whereas no significant relationship was found in diabetic CKD patients.

Current therapy to retard the progression of CKD has yielded disappointing outcomes despite aggressive control of BP. Parameters currently used to assess the adequacy of BP control need to be reassessed [22]. BP is fundamentally labile even in the normotensive state, and it is the intact auto-regulatory mechanisms that prevent the normally occurring BP fluctuations from target organs [23]. Therefore, if auto-regulation is impaired, there is greater transmission of such BP lability to target organs, and pressure exposed to these organs might not be normalized even if overall normal BP is achieved with antihypertensive therapy. In this context, the percentage of ABPM above threshold values has been termed as “BP load” and suggested to be a clinically useful parameter complementing quantification of average ABP levels, which might be an index of hemodynamic burden on the cardiovascular system in addition to the information carried by average ABP levels [24, 25]. We should identify CKD patients even with a significantly increased BP load so that special implementation can be used not only to lower BP levels but also to normalized BP load and prevent subclinical organ damage. Any patient with a higher BP load means that key organs such as the heart and kidney as well as large arteries are exposed to higher pressure for a longer duration even if the mean BP is normal. Patients with increased nighttime BP load means overloading the cardiovascular system, with the consequent negative impact on the heart and vascular structures. Consequently, a higher night-time BP load affects the heart, vasculature, and kidneys, boosting damage and increasing the risk of developing clinical events [26].

Diabetes mellitus is an established risk factor for coronary heart disease and ischemic stroke. It confers an approximate two fold increased risk for a wide range of vascular diseases compared with non-diabetic patients [27]. Diabetic patients have a significantly higher night-time systolic BP and reduced night-time variability of heart rate than those without diabetes [28]. Another fact that diabetic CKD patients have worse control of SBP, higher prevalence of a non-dipping pattern, and cardiovascular disease compared with non-diabetic CKD patients [29, 30]. All these data would lead to different results of ambulatory BP and TOD compared with non-diabetic patients. Our results showed different role of BP load in CKD patients with diabetic or non-diabetic: night-time SBP load correlated with LVMI, proteinuria in non-diabetic CKD patients without hypertension and correlated with LVMI, cIMT and eGFR in non-diabetic CKD patients with hypertension, while BP load did not refine this correlation based on the 24-h BP level in diabetic CKD patients. All these data suggested different role of BP load in diabetic and non-diabetic patients might be related to the complicated conditions in diabetic patients.

In our current study, the correlation coefficients between level and load were all >0.6, multiple regression analysis which include both level and load to assess the independent effect of a single variable may be misleading because of multicollinearity. To avoid the effect of multicollinearity, 24h-BP and load were included in model 1 and model 2 respectively by multivariable-adjusted analysis. In the next step, to investigate whether the correlations between BP load and TOD were over and beyond the 24-h BP level. We added significantly associated BP load to model 3 which already including the significantly associated 24-h BP and other covariables and computed the R squared change and p value.

This study had limitations that should be considered when evaluating our results. Firstly, because of the cross-sectional design of the study, we could not establish a causal relationship between BP load and TOD, but merely observe an association. Secondly, drug would not have affected the result of analyses since patients who received any antihypertensive drug in the previous month were excluded. However, we could not rule out the effect of chinese medicines. Thirdly, all CKD patients were admitted to our hospital division. Actually, these patients had severe proteinuria or severe renal damage, so some CKD patients with non-severe proteinuria or non-severe renal damage might have been omitted, which lead to some difference from general CKD patients. Therefore, further multi-center, larger-sample follow-up studies are necessary to explore the relationship between BP load and TOD.

In conclusion, our analyses showed that BP level and load were both independently associated with TOD. However, adding BP load to models already including BP level was able to additionally explain the variance of TOD in non-diabetic CKD patients. Our study provides important information that BP load might be an independent risk factor for cardiac, large vascular and renal damages in non-diabetic CKD patients, and we should undertake comprehensive assessment of BP in the management of non-diabetic CKD patients.
